# Pioglitazone for primary stroke prevention in Asian patients with type 2 diabetes and cardiovascular risk factors: a retrospective study

**DOI:** 10.1186/s12933-020-01056-x

**Published:** 2020-06-20

**Authors:** Yi-Chih Hung, Lu-Ting Chiu, Hung-Yu Huang, Da-Tian Bau

**Affiliations:** 1 Graduate Institute of Clinical Medical Science, China Medical University, Taichung, Taiwan; 2 Division of Endocrinology and Metabolism, Department of Medicine, China Medical University Hospital, Taichung, Taiwan; 3 Management Office for Health Data, China Medical University Hospital, Taichung, Taiwan; 4 College of Medicine, China Medical University, Taichung, Taiwan; 5 Department of Neurology, China Medical University Hospital, Taichung, Taiwan; 6 Terry Fox Cancer Research Laboratory, China Medical University Hospital, Taichung, Taiwan; 7 Department of Bioinformatics and Medical Engineering, Asia University, Taichung, Taiwan

**Keywords:** Pioglitazone, Stroke, Asian patient, Type 2 diabetes

## Abstract

**Background:**

Studies assessing the efficacy of pioglitazone solely for primary stroke prevention in Asian patients with type 2 diabetes mellitus (DM) and present multiple cardiovascular (CV) risk factors are rare. Thus, we aimed to assess the effect of pioglitazone on primary stroke prevention in Asian patients with type 2 DM without established CV diseases but with risk factors for CV diseases.

**Methods:**

Between 2000 and 2012, we enrolled patients aged ≥ 18 years, who were newly diagnosed with type 2 diabetes and had at least one of the following CV risk factors: hypertension and hyperlipidemia. Patients with a history of stroke and those using insulin or glucagon-like peptide-1 agonist for more than 3 months were excluded. Patients were divided into the pioglitazone and non-pioglitazone groups based on their receipt of pioglitazone during the follow-up period. Propensity-score matching (1:1) was used to balance the distribution of the baseline characteristics and medications. Follow-up was terminated upon ischemic stroke development, withdrawal from the insurance system, or on December 31, 2013, whichever occurred first. The overall incidence of new-onset ischemic stroke in the two groups was subsequently compared. The subgroup analyses of ischemic stroke were conducted using different baseline features. Additionally, the effect of pioglitazone exposure dose on the occurrence of ischemic stroke was evaluated. Chi square test, Student’s *t*-test, competing risk regression models, Kaplan–Meier method, and log-rank test were some of the statistical tests conducted.

**Results:**

A total of 13 078 patients were included in the pioglitazone and non-pioglitazone groups. Compared with patients who did not receive pioglitazone, those administered pioglitazone had a lower risk of developing ischemic stroke (adjusted hazard ratio: 0.78; 95% confidence interval: 0.62–0.95). The subgroup analyses defined by different baseline features did not reveal significant alterations in the observed effect of pioglitazone. Moreover, a significant decreasing trend in ischemic stroke risk with an increase in pioglitazone dose (p-value for trend = 0.04) was observed.

**Conclusion:**

Pioglitazone use decreased the risk of new-onset ischemic stroke in Asian patients with type 2 DM and CV risk factors.

*Trial registration number* CMUH104-REC2-115-CR4

## Background

Although the incidence of stroke has decreased in most regions, its incidence has increased in East Asia [1]. Stroke is the third leading cause of death in Taiwan, with ischemic stroke being the most common type [2]. Compared with the non-diabetic population, the risk of stroke is increased in patients with type 2 diabetes mellitus (DM) [3]. Additionally, it is an important contributor to stroke morbidity [2]. Pioglitazone is an oral glucose-lowering agent belonging to the drug class known as thiazolidinediones (TZD). Pioglitazone acts as an agonist of the peroxisome proliferator-activated receptor γ. Treatment with TZD has been demonstrated to reduce neuro-inflammation and improve the survival of neurons and glial cells [4, 5]. TZD therapy has also been shown to prevent or mitigate the progression of carotid intima–media thickness, a risk factor for ischemic stroke [6, 7]. Owing to this property, pioglitazone can exert protective effects on the cerebrovascular system. Currently, pioglitazone is generically available and cost-effective. As a result, it is a more affordable option for cerebrovascular protection. In a subgroup analysis of patients with type 2 DM and previous stroke in the PROactive trial [8], the rate of fatal or non-fatal stroke events was significantly lower in the pioglitazone group than the placebo group (hazard ratio: 0.53; event rate 5.6% in the pioglitazone group vs. 10.2% in the placebo group, 95% confidence interval: 0.34–0.85; number needed to treat = 22) [9]. In the IRIS study, which included patients with insulin resistance and recent stroke or transient ischemic attack, a lower incidence of stroke or myocardial infarction (MI) was observed in patients administered pioglitazone [10]. The Juntendo Stroke Prevention study in Insulin Resistance and Impaired glucose Tolerance (J-SPIRIT) study was another randomized trial consisting of 120 patients with impaired glucose tolerance or newly diagnosed DM in Japan, who had experienced a non-disabling ischemic stroke or aTIA [11]. Over a median follow-up period of 2.8 years, treatment with pioglitazone was not significantly associated with a lower risk of recurrent stroke (HR 0.62, 95%, CI 0.13–2.25, p = 0.49). Nevertheless, the number of enrolled patients in that study was too small. A meta-analysis of the above three trials demonstrated that treatment with pioglitazone in stroke patients with insulin resistance, prediabetes, and DM was associated with a significantly lower risk of recurrent stroke (HR 0.68, 95% CI 0.50–0.92, p = 0.01) [12]. Data from these studies provide strong evidence to support the use of pioglitazone for secondary stroke prevention, and it is possible that the results could be extended in the future to populations without a history of stroke [13]. Based on real-world data, the effect of pioglitazone on stroke varies according to the different clinical characteristics of patients and the interaction with other glucose-lowering agents [14–18]. A meta-analysis of randomized-controlled trials (RCTs) revealed that pioglitazone reduced the risk of stroke in patients with a history of established cardiovascular (CV) diseases [19]. However, as most patients with type 2 DM do not have established CV diseases, determining whether pioglitazone exerts cerebrovascular benefits in patients without established CV diseases, but present multiple risk factors, particularly those with a higher risk of ischemic stroke (e.g., patients of Asian descent), is crucial. Studies assessing the efficacy of pioglitazone solely for primary stroke prevention in Asian patients with type 2 DM and present multiple CV risk factors are rare.

Therefore, to investigate the effect of pioglitazone on primary stroke prevention in Asian patients without established CV diseases, but present risk factors for CV diseases, we conducted a population-based cohort study using the database of the Taiwan National Health Insurance (NHI) program.

## Methods

### Aim and design

To assess the effect of pioglitazone on primary stroke prevention in Asian patients with type 2 DM without established CV diseases but with risk factors for CV diseases, we opted to perform a retrospective study using claims data.

### Data source

The Taiwan National Health Insurance Research Database (NHIRD) contains the annual reimbursement claim data from the NHI program, which has been the universal health insurance system in Taiwan since 1996, covering approximately 99% of the Taiwanese population by 1998 [20]. The Longitudinal Health Insurance Database (LHID), which is a subset of the NHIRD, includes historical claims data for one million subjects who were randomly sampled from the entire insured population from 1996 to 2000. Before the release of data for research, all personal identification data in the LHID were de-identified to protect the privacy of patients by the National Health Research Institute via an anonymized number system, which linked each claimant’s demographic information to the LHID. The International Classification of Diseases, Ninth Revision, Clinical Modification (ICD-9-CM) is used by the NHIRD to categorize disease diagnoses based on outpatient and inpatient data.

### Ethical approval

This study was approved by the Ethics Review Board of China Medical University (CMUH104-REC2-115-CR4), who waived the need for informed consent based on the retrospective design of the study.

### Study population

**Fig. 1:**
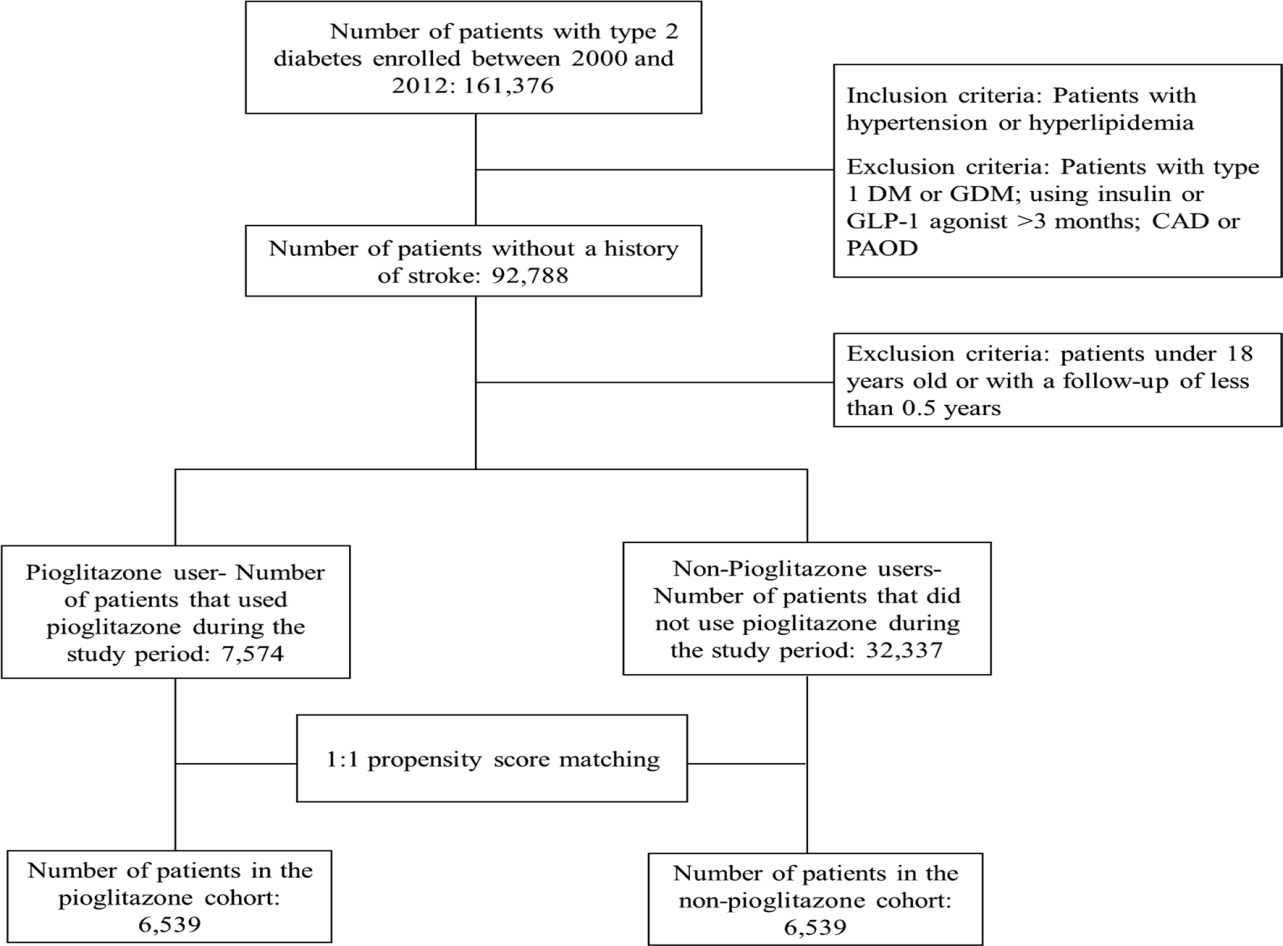
Flow chart for cohort selection

Between 2000 and 2012, we enrolled patients aged ≥ 18 years, who were newly diagnosed with type 2 diabetes (ICD-9-CM codes 250) and had at least one of the following CV risk factors: hypertension (HTN)(ICD-9-CM 401–405) and hyperlipidemia (ICD-9-CM 272). We excluded patients diagnosed with stroke (ICD-9-CM 430-438), type 1 DM (ICD-9-CM 250. × 1 and 250. × 3), and gestational diabetes mellitus (GDM) (ICD-9-CM 648.83), using insulin or glucagon-like peptide-1 (GLP-1) agonist for more than 3 months; coronary artery disease (CAD) (ICD-9-CM 414.00–414.05, 414.8, and 414.9); and peripheral artery occlusive disease (PAOD) (ICD-9-CM 440.0, 440.2, 440.3, 440.8, 440.9, 443, 444.0, 444.22, 444.8, 447.8, and 447.9); or with a follow-up period of < 0.5 years. The index day was defined as the date of the first prescription of pioglitazone. The subjects were divided into the pioglitazone and non-pioglitazone groups according to the receipt of pioglitazone during the follow-up period (Fig. 1). By propensity score (PS) matching, each patient without pioglitazone treatment was matched for one pioglitazone-treated patient by age; sex; presence or absence of heart failure (HF) (ICD-9-CM codes 428), arrhythmia (ICD-9-CM codes 427), chronic renal disease (CKD) (ICD-9-CM codes 585), HTN (ICD-9-CM codes 401–405), and hyperlipidemia (ICD-9-CM 272); and the administration of anti-hypertensive medication, lipid-lowering agents, anti-platelet agents, and oral glucose-lowering agents. Follow-up was terminated upon hospitalization for ischemic stroke (which was ascertained by the ICD-9-CM codes 433–435 in the first position of the hospital discharge diagnoses), a withdrawal from the insurance system, or on December 31, 2013, whichever occurred first. The overall incidence of new-onset ischemic stroke in the two groups was subsequently compared.

### Statistical analysis

The Chi square test and Student’s *t*-test were used to compare the differences in the categorical variables and continuous variables, respectively, between the groups. The incidence rate of an event was estimated using the number of events and person-years. Because all-cause mortality is a competing risk of the primary outcome, we considered all-cause mortality during the follow-up period as a competing risk. The hazard ratio (HR) and 95% confidence interval (CI) for the risk of events were estimated using univariate and multivariate competing risk regression models. The multivariate model was adjusted for age, sex, comorbidities, and the medications listed in Table 1. Subgroup analyses of new-onset ischemic stroke were conducted with 10 pre-specified subgroup variables, namely, age, sex, HF, arrhythmia, CKD, HTN, hyperlipidemia, number of CV risk factors, number of anti-hypertensive agents, and number of glucose-lowering agents. We used the defined daily dose (DDD) per year to quantify the average dose of pioglitazone. Based on DDD, we established the following four categories of dose exposure: no exposure, low dose exposure (< 100 DDD per year), intermediate dose exposure (100–250 DDD per year), and high dose exposure (> 250 DDD per year) to evaluate the effect of exposure dose on the occurrence of ischemic stroke. The cumulative incidence of new-onset ischemic stroke was assessed using the Kaplan–Meier method and differences between groups were determined using a log-rank test. All statistical analyses were performed using SAS statistical software (Version 9.4 for Windows; SAS Institute, Inc., Cary, NC, USA). Statistical significance was defined at p < 0.05.

**Table 1:** Baseline demographic characteristics of patients with DM treated with and without pioglitazone

Characteristics	Before PS matching				p-value	After PS matching				p-value
	Non-pioglitazone user n = 32,337		Pioglitazone user n = 7574			Non-pioglitazone user n = 6539		Pioglitazone user n = 6539		
	N	%	n	%		N	%	n	%	
Age, years										
< 55	12,160	37.60	2810	37.10	0.42	2302	35.2	2338	35.75	0.51
55–65	8960	27.71	2463	32.52	< 0.0001	2085	31.89	2126	32.51	0.44
≥ 65	11,217	34.69	2301	30.38	< 0.0001	2152	32.91	2075	31.73	0.15
Mean ± SD	59.48 ± 13.36	59.09 ± 11.31	0.001	59.74 ± 12.32	59.52 ± 11.51	0.29				
Gender										
Female	17,068	52.78	3801	50.18	< 0.0001	3250	49.70	3242	49.58	0.89
Male	15,269	47.22	3773	49.82	< 0.0001	3289	50.30	3297	50.42	0.89
Comorbidity										
Heart failure	1920	5.94	326	4.30	< 0.0001	306	4.68	296	4.53	0.68
Arrhythmia	4686	14.49	768	10.14	< 0.0001	690	10.55	695	10.63	0.89
Chronic kidney disease	3621	11.20	788	10.40	0.05	665	10.17	681	10.41	0.65
Vascular risk factor										
Hypertension	21,589	66.76	5677	74.95	< 0.0001	4863	74.37	4843	74.06	0.69
Hyperlipidemia	21,387	66.14	5956	78.64	< 0.0001	5003	76.51	5022	76.80	0.69
Number of risk factors										
1	18,750	57.98	3136	41.40	< 0.0001	2899	44.33	2848	43.55	0.36
2	13,587	42.02	4438	58.60	< 0.0001	3640	55.67	3691	56.45	0.36
Drug use										
Hypertensive agents										
ACEI	13,087	40.47	40.16	53.02	< 0.0001	3382	51.72	3386	51.78	0.94
ARB	7213	22.31	2727	36.00	< 0.0001	2257	34.52	2263	34.61	0.91
α-Blocker	6744	20.86	1616	21.34	0.35	1418	21.69	1418	21.69	1.00
β-Blocker	19,379	59.93	4498	59.39	0.39	3873	59.23	3893	59.54	0.72
CCB	13,766	42.57	3569	47.48	<0.0001	3154	48.23	3087	47.21	0.24
Diuretics	12,474	38.58	3045	40.20	0.009	2702	41.32	2656	40.62	0.41
Others	2736	8.46	538	7.10	0.0001	463	7.08	475	7.26	0.68
Number of hypertensive agents										
≤ 1	10,911	33.74	2096	27.67	< 0.0001	1809	27.66	1834	28.05	0.62
2	7432	22.98	1561	20.61	< 0.0001	1356	20.74	13.42	20.52	0.76
≥ 3	13,994	43.28	3917	51.72	< 0.0001	3374	51.6	3363	51.43	0.84
Hypolipidemic agents										
Statin	10,366	32.06	4506	59.49	< 0.0001	3664	56.03	3664	56.03	1.00
Initial statin therapy										
High intensity*	32	0.31	16	0.36	0.01	13	0.35	13	0.35	1.00
Moderate intensity**	5418	52.27	2677	59.41	< 0.0001	1936	52.84	2153	58.76	< 0.0001
Low intensity***	4916	47.42	1813	40.24	< 0.0001	1715	46.81	1498	40.88	< 0.0001
Fibrate	6984	21.6	2805	37.03	< 0.0001	2304	35.23	2282	34.90	0.69
Others	7621	23.57	2881	38.04	< 0.0001	2397	36.66	2383	36.44	0.80
Anti-platelet agents										
Aspirin	17,438	53.93	4354	57.49	< 0.0001	3735	57.12	3723	56.94	0.83
Warfarin	320	0.99	50	0.66	0.007	44	0.67	47	0.72	0.75
Clopidogrel	575	1.78	178	2.35	0.001	149	2.28	159	2.43	0.56
Others	2644	8.18	636	8.40	0.52	538	8.23	551	8.43	0.68
Oral antidiabetic agents										
Metformin	10,103	31.24	6588	86.98	< 0.0001	5610	85.79	5557	84.98	0.19
Sulfonylureas	12,478	38.59	7111	93.89	< 0.0001	6155	94.13	6076	92.92	0.01
DPP4 inhibitors	569	1.76	672	8.87	0.001	465	7.11	499	7.63	0.26
Alpha-glucosidase inhibitor	1977	6.11	2521	33.28	< 0.0001	1515	23.17	1685	25.77	0.001
Glinide	1338	4.14	1430	18.88	< 0.0001	925	14.15	1034	15.81	0.01
Number of oral antidiabetic agents										
≤ 1	23,544	72.81	893	11.79	< 0.0001	896	13.70	893	13.66	0.94
2	6553	20.26	3418	45.13	< 0.0001	3561	54.46	3360	51.38	0.0004
3	1723	5.33	2411	31.83	< 0.0001	1568	23.98	1808	27.65	< 0.0001
≥ 4	517	1.60	852	11.25	< 0.0001	514	7.86	478	7.31	0.23
Follow-up duration, year	5.25 ± 3.19	4.36 ± 2.36	<0.0001	4.19 ± 2.64	4.45 ± 2.39	< 0.0001				

Data are presented as n (%) or mean ± SD

Number of oral glucose-lowering agents used including metformin, sulfonylureas, DPP4 inhibitors, alpha-glucosidase inhibitor, and glinide

*PS* propensity score, *ACEI* angiotensin-converting-enzyme inhibitor, *ARB* angiotensin receptor blockers, *CCB* calcium channel blockers

* High-intensity statins: atorvastatin ≥ 40 mg/day, or rosuvastatin ≥ 20 mg/day

** Moderate-intensity statins: 10 mg/day ≤ atorvastatin < 40 mg/day, 5 mg/day ≤ rosuvastatin < 20 mg/day, 20 mg/day ≤ simvastatin, pravastatin ≥ 40 mg/day, lovastatin ≥ 40 mg/day and fluvastatin ≥ 80 mg/day

*** Low-intensity statins: atorvastatin < 10 mg/day, rosuvastatin < 5 mg/day, simvastatin < 20 mg/day, pravastatin < 40 mg/day, lovastatin < 40 mg/day, and fluvastatin < 80 mg/day

## Results

A total of 13,078 patients treated with and without pioglitazone were matched in a 1:1 ratio. The demographic characteristics of the two cohorts were almost similar (Table 1). Most patients were aged < 65 years and 50% were males. Approximately 4%, 10%, and 10% of the patients in both cohorts had HF, arrhythmia, and CKD, respectively. Additionally, approximately 74% and 76% of patients in the two groups had HTN and hyperlipidemia, respectively. Forty-four percent of the patients had one CV risk factor, whereas 56% had had two CV risk factors. The number of patients treated with angiotensin-converting-enzyme inhibitor (ACEI), angiotensin receptor blockers (ARB), α-blocker, β-blocker, calcium channel blockers (CCB), diuretics, and other anti-hypertensive agents was similar between the two groups. Approximately 28%, 20%, and 51% of patients were treated with ≤ 1, 2, and ≥ 3 anti-hypertensive agents, respectively. Approximately 56% of the patients were treated with statin and less than 1% of the patients in both groups used high-intensity statin. More patients in the pioglitazone cohort used moderate-intensity statin (pioglitazone cohort: 58.76%, non-pioglitazone cohort: 52.84%; p < 0.01), whereas, more patients in the non-pioglitazone cohort used low-intensity statin (pioglitazone cohort: 46.81%, non-pioglitazone cohort: 40.88%; p < 0.01). Approximately 35% of the patients used fibrate, 36% of whom were also treated with other cholesterol-lowering agents. Approximately 57% of the patients in both groups used aspirin. Less than 1%, 2%, and 8% of the patients in both groups used warfarin, clopidogrel, and other anti-platelet agents, respectively. More patients in the non-pioglitazone cohort used sulfonylureas (SU) (pioglitazone cohort: 92.92%, non-pioglitazone cohort: 94.13%; p = 0.01) and more patients in the pioglitazone cohort used α-glucosidase inhibitor (pioglitazone cohort: 25.77%, non-pioglitazone cohort: 23.17%; p < 0.01) and glinide (pioglitazone cohort: 15.81%, non-pioglitazone cohort: 14.15%; p = 0.01). However, the number of patients who used metformin and dipeptidyl peptidase 4 (DPP4) inhibitors was similar between the two groups. Approximately 13% of the patients used no more than one glucose-lowering agents and 7% of the patients in both groups used more than four glucose- lowering agents. More patients in the non-pioglitazone cohort used two glucose-lowering agents (pioglitazone cohort: 51.38%, non-pioglitazone cohort: 54.46%; p < 0.01), whereas more patients in the pioglitazone cohort used three glucose-lowering agents (pioglitazone cohort: 27.65%, non-pioglitazone cohort: 23.98%; p < 0.01). The mean follow-up duration was ~ 4 years in both cohorts, but it was longer in the pioglitazone cohort than in the non-pioglitazone cohort (4.45 ± 2.39 years vs. 4.19 ± 2.64 years; p < 0.01).

As shown in Table 2, the overall incidence of ischemic stroke was 29 268 per 1000 person-years in the pioglitazone cohort, a value lower than that found in the non-pioglitazone cohort (27 682 per 1000 person-years), with an adjusted hazard ratio (aHR) of 0.78 (95% CI 0.62–0.95, p= 0.03).

**Table 2:** Hazard ratios and 95% confidence intervals of ischemic stroke owing to pioglitazone use

Variables	Ischemic stroke (n = 306)			Crude HR (95% CI)	p-value	Adjusted HR (95% CI)	p-value
	Event	PY	IR				
Pioglitazone							
No	170	27,682	6.14	1 (reference)		1 (reference)	
Yes	136	29,268	4.65	0.75 (0.60–0.90)**	0.009	0.78 (0.62–0.95)*	0.03

HR adjusted for age, sex, heart failure, arrhythmia, chronic kidney disease, hypertension, hyperlipidemia, hypertensive agents, lipid-lowering agents, anti-platelet agents, and oral glucose-lowering agents

*PY* person-years, *IR* incidence rate, per 1000 person-years, *HR* hazard ratio, *CI* confidence interval, *PY* person-year, *IR* incidence rate, per 1000 person-years, *HR* hazard ratio, *CI* confidence interval

* p < 0.05, ** p < 0.01

The subgroup analyses defined by the different baseline features did not disclose any significant alterations in the observed effect of pioglitazone (Table 3; all p-values for interaction > 0.05).

**Table 3:** Hazard ratios and 95% confidence intervals of ischemic stroke stratified by age, sex, and comorbidities between the pioglitazone and non-pioglitazone groups

Variables	Non-pioglitazone user (n = 6539)			Pioglitazone user (n = 6539)			Crude HR (95% CI)	Adjusted HR (95% CI)	p for interaction
	Event	PY	IR	Event	PY	IR			
All	170	27,682	6.14	136	29,268	4.65	0.75 (0.60–0.90)**	0.78 (0.62–0.95)*	
Age group (years)									0.11
< 45	46	10,445	4.40	23	10,857	2.12	0.48 (0.29–0.79)**	0.50 (0.30–0.82)**	
45–64	52	8663	6.00	45	9659	4.66	0.77 (0.52–1.16)	0.76 (0.51–1.14)	
≥ 65	72	8573	8.40	68	8752	7.77	0.90 (0.65–1.29)	0.89 (0.64–1.25)	
Gender									0.58
Female	73	14,124	5.17	63	14,981	4.21	0.81 (0.57–1.13)	0.85 (0.30–1.20)	
Male	97	13,557	7.15	73	14,287	5.11	0.71 (0.52–0.96)*	0.68 (0.20–0.92)*	
Comorbidity									0.16
Heart failure									
No	156	26,555	5.87	130	28,034	4.64	0.78 (0.62–0.99)*	0.79 (0.2–1.01)	
Yes	14	1126	12.43	6	1234	4.86	0.38 (0.15–0.96)*	0.38 (0.14–1.03)	
Arrhythmia									0.63
No	152	24,900	6.10	124	26,350	4.71	0.76 (0.60–0.95)*	0.76 (0.60–0.97)*	
Yes	18	2781	6.47	12	2918	4.11	0.67 (0.32–1.41)	0.89 (0.41–1.95)	
Chronic kidney disease									0.57
No	154	24,998	6.16	126	26,500	4.75	0.76 (0.61–0.97)*	0.77 (0.61–0.98)*	
Yes	16	2683	5.96	10	2768	3.61	0.60 (0.27–1.32)	0.51 (0.22–1.21)	
Hypertension									0.59
No	28	7737	3.62	25	8027	3.11	0.87 (0.51–1.51)	0.87 (0.50–1.52)	
Yes	142	19,944	7.12	111	21,241	5.23	0.72 (0.57–0.93)*	0.74 (0.57–0.95)*	
Hyperlipidemia									0.54
No	56	7518	7.45	46	7388	6.23	0.83 (0.56–1.24)	0.83 (0.55–1.24)	
Yes	114	20,163	5.65	90	21,880	4.11	0.71 (0.54–0.94)**	0.72 (0.54–0.96)*	
Number of risk factor									0.36
1	72	13,348	5.39	61	13,391	4.56	0.84 (0.60–1.19)	0.85 (0.60–1.20)	
2	98	14,333	6.84	75	15,877	4.72	0.68 (0.50–0.92)*	0.70 (0.52–0.95)*	
Number of hypertensive agents									0.23
≤ 1	44	8820	4.99	39	9002	4.33	0.87 (0.57–1.35)	0.86 (0.55–1.35)	
2	36	6058	5.94	35	6131	5.71	0.95 (0.59–1.51)	0.93 (0.57–1.50)	
≥ 3	90	12,802	7.03	62	14,134	4.39	0.61 (0.44–0.85)**	0.64 (0.46–0.89)**	
Number of oral antidiabetic agents									0.14
≤ 1	39	4666	8.36	17	4563	3.73	0.44 (0.25–0.79)**	0.41 (0.23–0.75)**	
2	84	15,397	5.46	74	15,125	4.89	0.46 (0.89–1.21)	0.91 (0.66–1.25)	
3	33	6052	5.45	37	7873	4.70	0.84 (0.52–1.25)	0.89 (0.55–1.42)	
≥ 4	14	1566	8.94	8	1706	4.69	0.52 (0.22–1.24)	0.56 (0.22–1.43)	

HR adjusted for age, sex, heart failure, arrhythmia, chronic kidney disease, hypertension, hyperlipidemia, hypertensive agents, lipid-lowering agents, anti-platelet agents, and oral glucose-lowering agents

* p < 0.05, ** p < 0.01

Compared with non-pioglitazone users, individuals exposed to low-, intermediate-, or high-dose pioglitazone did demonstrate an association, with a 0.79-fold (adjusted HR 0.79, 95% CI 0.58–1.06), 0.74-fold (adjusted HR 0.74, 95% CI 0.53–0.98), and 0.66-fold (adjusted HR 0.66, 95% CI 0.46–0.87) decrease in the risk of ischemic stroke, respectively (Table 4). Moreover, there was a significant decreasing trend of ischemic stroke risk with the increase in pioglitazone dose (p-value for trend = 0.04).

**Table 4:** Hazard ratios and 95% confidence intervals of ischemic stroke associated with the defined daily doses of pioglitazone

Variables	Ischemic stroke (n = 306)			Crude HR (95% CI)	Adjust HR (95% CI)
	Event	PY	IR		
Pioglitazone, DDD (per year)					
0	170	27,682	6.14	1 (reference)	1 (reference)
< 100	58	12,093	4.80	0.77 (0.57–1.03)	0.79 (0.58–1.06)
100–250	41	8935	4.59	0.74 (0.52–0.95)*	0.74 (0.53–0.98)*
> 250	37	8239	4.49	0.63 (0.45–0.84)**	0.66 (0.46–0.87)*
p for trend				0.02	0.04

HR adjusted for age, sex, heart failure, arrhythmia, chronic kidney disease, hypertension, hyperlipidemia, hypertensive agents, lipid-lowering agents, anti-platelet agents, and oral glucose-lowering agents

* p < 0.05, ** p < 0.01

**Fig. 2:**
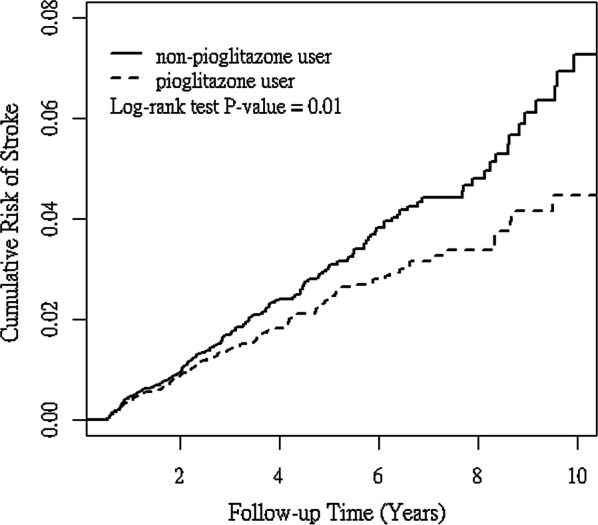
Cumulative incidence of ischemic stroke in pioglitazone (solid line) and non-pioglitazone (dashed line) users

As shown in Fig. 2, the cumulative incidence of ischemic stroke was significantly lower in the pioglitazone cohort than in the non-pioglitazone cohort (log-rank test, p= 0.01).

## Discussion

Our findings revealed that the use of pioglitazone was associated with a decreased risk of ischemic stroke among Asian patients with type 2 diabetes but present risk factors for CV diseases. To the best of our knowledge, this is the first study to assess the efficacy of pioglitazone for primary stroke prevention alone in Asian patients with type 2 DM and no established CV diseases, but present risk factors for CV diseases.

Although pioglitazone is now generically available and more cost-effective than a sodium-glucose cotransporter 2 (SGLT2) inhibitor or a GLP-1 receptor agonist for CV protection, more clinical data may be needed to support the protective effects of pioglitazone against stroke in patients with type 2 DM and no established CV diseases. To date, RCTs assessing the effect of pioglitazone on primary stroke prevention in these patients are lacking. In 2006, the CHICAGO study (Carotid Intima-Media Thickness in Atherosclerosis Using Pioglitazone) revealed that pioglitazone slowed the progression of CIMT over an 18-month treatment period in patients with type 2 DM and no prior CV disease compared with glimepiride [21]. In 2017, the Thiazolidinediones or Sulfonylureas Cardiovascular Accidents Intervention Trial (TOSCA.IT) was conducted with patients aged 50–75 years with inadequately controlled type 2 DM with metformin monotherapy from 57 diabetes clinics in Italy [22]. Based on the findings, only 11% of the patients had baseline CV disease and 1–2% of subjects had a previous stroke. Nonetheless, the incidence of CV events, including non-fatal stroke, was similar to that with SUs and pioglitazone as add-on treatments to metformin [22]. Considering the large heterogeneity of patients with type 2 DM and the need for a personalized approach, a recent post hoc analysis of TOSCA.IT revealed that men with a urine albumin/creatinine ratio greater than 9 mg/g and body mass index > 28.8 kg/m^2^ presented benefits owing to pioglitazone at an HR of 0.48 (95% CI, 0.25–0.76) compared with SUs [23]. In Asian patients with type 2 DM without prior CV diseases, the real-world data demonstrated controversial stroke-protective effects of pioglitazone. Chan et al. [24] demonstrated that compared with sulfonylurea plus metformin, pioglitazone added to metformin therapy may have fewer major CV events, including ischemic stroke in patients with type 2 DM. However, another real-world study conducted by Lu et al. did not reveal the protective effects of pioglitazone on ischemic stroke prevention [25]. These conflicting results may be due to the different clinical characteristics of patients and an interaction with other glucose-lowering agents. In our study, we excluded patients who used insulin or GLP-1 agonist for more than 3 months and included patients with at least one or more CV risk factors. Moreover, “patients treated with pioglitazone” in our study included those who took pioglitazone during the follow-up period instead of baseline pioglitazone treatment. After PS matching to match all baseline characteristics and adjusting for potential confounders, our study revealed that the use of pioglitazone was associated with a decreased risk of ischemic stroke. Thus, our data provided evidence that pioglitazone could be administered for the primary prevention of ischemic stroke in Asian patients with type 2 DM without prior CV diseases, but present risk factors for CV diseases. Defining such a group of patients with a different likelihood of benefitting from pioglitazone treatment represents an important clinical need. Furthermore, this result was similar to the findings of a recent meta-analysis [26] that evaluated the effect of pioglitazone on the primary and secondary prevention of CV diseases in patients “with or at a high risk” of type 2 DM. In this meta-analysis of 26 RCTs with 19 645 participants, although a greater reduction in non-fatal myocardial infarction, non-fatal stroke, or CV death was noted in patients with a history of established CV diseases than those without, the subgroup differences between the primary and secondary prevention were not statistically significant (p-value for subgroup heterogeneity > 0.05) [26].

Previously, the relationship between pioglitazone dose and its protective effect on primary stroke prevention in patients with type 2 DM was unclear. A post hoc analysis of the IRIS study revealed that the HR of recurrent ischemic stroke could be lower in patients in the subgroup with a pioglitazone adherence ≥ 80% than in those in the intention-to-treat analysis [27]. In our study, there was a significant decreasing trend of ischemic stroke risk with the increasing dose of pioglitazone (p-value for trend = 0.04). However, it is noteworthy that the use of pioglitazone is difficult to be accepted among measures for the prevention of stroke. This might have derived from the fear of dose-related adverse effects of the drug, such as weight gain and fluid retention [13]. Combination therapy with pioglitazone plus an SGLT2 inhibitor might reduce the frequency of weight gain or edema [28] and beneficial effects of pioglitazone on stroke could additively improve CV outcome when combined with SGLT2 inhibitors [29].

Our study had several strengths. First, the use of an administrative database prevented the under reporting of medical visits. Second, its national population-based design enabled our study to be highly representative of the general population and prevented a selection bias. Third, the risk of misclassification by excluding patients who might have had other types of diabetes (patients administered insulin for more than 3 months) was reduced. Fourth, PS matching was employed to match almost all baseline characteristics and adjust for potential confounders during the analysis of the risk of ischemic stroke between pioglitazone and non-pioglitazone users.

Nevertheless, this study had some limitations. First, because this was an observational study, it may be affected by bias and the poor control of confounding factors. Second, the identities of patients were encrypted for privacy and data security reasons. As a result, we could not contact patients to discuss their use of pioglitazone. Third, several potential confounding factors, such as blood pressure (BP), serum glucose level, and lipid panel, were not included in the database. Nonetheless, the number of antihypertensive drugs and oral glucose-lowering agents, and the intensity of initial stain therapy were PS matched to mitigate the bias associated with different levels of BP, blood sugar, and serum lipid between the two groups. Fourth, although experts from the NHI program regularly review randomly selected medical records to confirm the diagnosis from all hospitals, bias may still arise due to miscoding. However, the diagnoses in the NHIRD have previously been validated [30, 31]. Finally, as our study included only Taiwanese patients who may have been at a greater risk of developing ischemic stroke due to their Asian descent, our results may not be applicable to other populations.

## Conclusions

In conclusion, the use of pioglitazone was associated with a decreased risk of new-onset ischemic stroke among Asian patients with type 2 diabetes and no established CV diseases, but present risk factors for CV diseases. Moreover, there was a significant decreasing trend ischemic stroke risk with the increase in pioglitazone dose. Further studies are thus required to determine the clinical relevance of pioglitazone on the primary prevention of stroke in patients with type 2 DM.

## Data Availability

The Taiwan National Health Insurance Research Database (NHIRD) collects the annual reimbursement claim data from the National Health Insurance program, which has been the universal health insurance system in Taiwan since 1996 (by 1998, the program covered almost 99% of the Taiwanese population) [20].

## Abbreviations

DM: Diabetes mellitus
TZD: Thiazolidinediones
MI: Myocardial infarction
RCTs: Randomized-controlled trial
CV: Cardiovascular
NHI: National Health Insurance
NHIRD: National Health Insurance Research Database
LHID: Longitudinal Health Insurance Database
ICD-9-CM: International Classification of Diseases, Ninth Revision, Clinical Modification
HTN: Hypertension
GDM: Gestational diabetes mellitus
GLP-1: Glucagon-like peptide-1
CAD: Coronary artery disease
PAOD: Peripheral artery occlusive disease
PS: Propensity score
HF: Heart failure
HR: Hazard ratio
CI: Confidence interval
DDD: Defined daily dose
CCB: Calcium channel blockers
SU: Sulfonylurea
DPP4: Dipeptidyl peptidase 4
SGLT2: Sodium-glucose cotransporter 2
TOSCA.IT: Thiazolidinediones or Sulfonylureas Cardiovascular Accidents Intervention Trial
BP: Blood pressure
